# Neuroprotective Effect of *Otostegia limbata* Against PTZ-Induced Mice Model of Epilepsy by Attenuated Expression of p-NFκB and TNF-α

**DOI:** 10.3389/fnins.2022.779681

**Published:** 2022-03-22

**Authors:** Farhana Amin, Sobia Tabassum, Sadia Sarwar, Rahmatullah Qureshi, Muhammad Sohaib Khalid, Naveeda Riaz, Wahidah H. Al-Qahtani, Iram Murtaza

**Affiliations:** ^1^Department of Biological Sciences, International Islamic University, Islamabad, Pakistan; ^2^Department of Pharmacognosy, Riphah Institute of Pharmaceutical Sciences, Faculty of Pharmaceutical Sciences, Riphah International University, Islamabad, Pakistan; ^3^Department of Botany, PMAS-Arid Agriculture University Rawalpindi, Rawalpindi, Pakistan; ^4^Department of Food Sciences and Nutrition, College of Food and Agriculture Sciences, King Saud University, Riyadh, Saudi Arabia; ^5^Department of Biochemistry and Molecular Biology, Quaid-I-Azam University, Islamabad, Pakistan

**Keywords:** neuroprotective effect, *O. limbata*, antioxidant potential, inflammatory cytokines, ELISA

## Abstract

Epilepsy is a chronic neurological disorder characterized by recurrent unprovoked *seizures.* Currently available antiepileptic drugs have severe side effects and do not offer complete cure. Herbal remedies have been used for centuries to treat many neurodegenerative disorders. *Otostegia limbata* L. belongs to the largest and medicinally important family Lamiaceae and is distributed in hilly areas of Pakistan. This study was designed to assess the antioxidant, anti-inflammatory, and anticonvulsant potential of *O. limbata.* The methanolic extract showed significant antioxidant activity assessed by (1,1-diphenyl 2-picrylhydrazyl) free-radical scavenging assay, nitric oxide scavenging, and iron chelation antioxidant assays. The methanolic extract was evaluated for its anticonvulsant effect, employing the pentylenetetrazole (PTZ)-induced mice model of epilepsy. Three different doses of *O. limbata* (100, 200, and 300 mg/kg) were administered orally 30 min before PTZ [50 mg/kg, intraperitoneal (i.p.)] injection, while diazepam was used as a positive control. The extract at 300 mg/kg significantly decreased the duration and increased the latency of the PTZ-induced seizures. The expression of inflammatory cytokines tumor necrosis factor α (p-TNF-α) and phosphorylated transcription factor nuclear factor kappa B (p-NF-κB), in the cortex and hippocampus of the brains of treated mice were analyzed through enzyme-linked immunosorbent assay and western blot analysis. The morphological changes and number of surviving neurons were recorded through hematoxylin and eosin staining. The seizure score and survival rate of the treated group showed considerable differences as compared to the PTZ group. TNF-α and p-NF-K b expression were downregulated as compared to the PTZ group. The anticonvulsant effect may be the outcome of the antioxidant potential and high levels of phenols and flavonoids detected in the methanolic plant extract through Fourier transform infrared spectrophotometer and gas chromatography–mass spectrometry analysis.

## Introduction

Epilepsy is the most serious and life-menacing neurological disorder distributed among 50 million people of the world (Visweswari et al., 2010). Due to the lack of proper epidemiological studies in Pakistan, it is estimated that the prevalence of epilepsy is 9.99/1,000 persons (Siddiqui et al., 2015). Epilepsy can be distinguished by recurrent unprovoked seizures and unexpected transient behavioral changes caused by an abnormal, synchronous rhythmic sacking of a set of brain neurological cells. Many studies suggest that an imbalance between inhibitory and excitatory conductance leads to seizures in normal brain tissue (Scharfman, 2007). Currently available antiepileptic drugs have severe side effects and do not cure effectively. Herbal remedies are being used for centuries to treat many disorders including neurodegenerative ailments.

Epilepsy has a multifactorial pathoetiological origin. Oxidative stress plays a key role in the initiation and progression of neurodegenerative diseases (Kasote et al., 2015). Free radicals produced during normal cellular metabolism generate oxidative stress (Phaniendra et al., 2015). It is now evident that mitochondrial oxidative stress plays a significant role in inducing epilepsy and other neurodegenerative diseases (Shekh-Ahmad et al., 2019).

The nuclear factor-kappa B (NF-kB) transcriptional complex predominantly regulates transcription-based cellular changes in brain cells by the upregulation of target genes. The NF-kB signaling pathway is very crucial in inflammation (Chen et al., 2018). TNF-α, an inflammatory cytokine produced in response to activated NF-kB, upregulates neurodegeneration (Scorza et al., 2018). TNF-α is produced by many cells, including macrophages and neuronal cells, in response to infections and inflammation. Neuroinflammation in the neuronal tissue upregulates the production of TNF-α in the brain, which enhances convulsion. TNF-α regulates glutamate receptor recruitment, and TNF receptors 1 (TNFR1) and 2 (TNFR2) can therefore stimulate neurons by raising glutamate levels in synaptic clefts. Prior research using the kindling model of epileptogenesis found a link between TNF-α. About 70–80% of epileptic patients are able to be commonly treated with modern anticonvulsant drugs that prevent from or lessen the number of seizure attacks. However, 30% of epileptic patients suffer from uncontrolled seizures even when they are using available drugs. There is an increasing need for searching for new drugs with a more effective and safer profile. Moreover, the adverse effects associated with anticonvulsants limit their use in patients (Ishola et al., 2013). *O. limbata* L. belongs to the medicinally important family Lamiaceae. The members of the Lamiaceae family are distributed all over the world. The genus Otostegia consists of 33 species, among which three species are found in Pakistan including *O. limbata*, *O. persica*, and *O. aucheri* (Khan and Syed, 2013). *O. limbata* is extensively utilized by traditional practitioners for several ailments. The reported actions include anti-ulcer, antidepressant, sedative, anxiolytic, and anti-inflammatory for eye inflammation, antibacterial, antioxidant, hemagglutination, and cholinesterase inhibition for Alzheimer’s treatment (Yu et al., 1999; Truelove et al., 2016; Vuong et al., 2016). *O. persica* has been reported for anticonvulsant activity that supports the ethnomedicinal claims of the use of the plant in the management of seizure (Ansari et al., 2014). *O. limbata* is also used for the treatment of many diseases, but to the best of our knowledge, this is the first report of the anticonvulsant activity of *O. limbata*.

## Materials and Methods

### Collection of Plant Material

**FIGURE 1:**
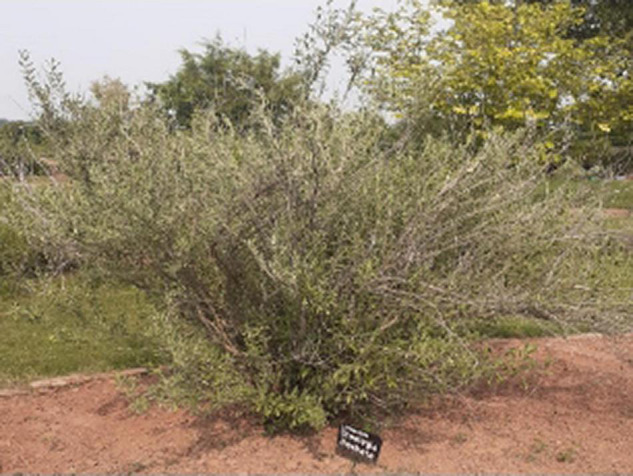
*Otostegia limbata* plant.

*Otostegia limbata* was collected from Islamabad, Pakistan (Figure 1). The plant was identified by taxonomist Dr. Rahmatullah Qureshi from the Department of Botany Arid Agriculture University Rawalpindi and assigned a voucher number (PMAS 390).

### Extract Preparation

The whole plant was washed thoroughly under tap water, shade-dried, and ground to a fine powder. The *O. limbata* extract was prepared by using the maceration method (Sasidharan et al., 2011). About 100 g of plant powder was soaked in 300 ml of methanol and kept on the shaker for 1 week. After shaking, the mixture was filtered by muslin cloth, followed by Whatman Filter Paper No. 1. The residues were kept for drying, and the dried extract was weighed, and% yield was calculated. The dried extract was subjected to fractional distillation using different solvents including n-hexane, chloroform, ethyl acetate, and butanol. All the fractions were dried in a rotary evaporator; their% yield was calculated and stored at 4°C for further use.

### Percentage Yield

The percentage yield of the crude extract was calculated by the formula mentioned below.


Percentage⁢yield=⁢(Crude⁢extract⁢weight/Dry⁢plant⁢weight)*100.

### Antioxidant Assays of Plant Extract

#### DPPH (1, 1-Diphenyl 2-Picrylhydrazyl) Free-Radical Scavenging Assay

The free-radical scavenging activity of the crude methanolic *O. Limbata* extract was evaluated by using the modified Brand-Williams et al. (1995) method. The bleaching ability of DPPH can be measured with the help of a spectrophotometer. The reaction mixture, composed of crude methanolic extracts of *O. limbata* and methanol solution of DPPH was incubated at room temperature for 30 min by using positive (ascorbic acid) and negative (DPPH solution) control. The absorbance was measured at 517 nm. Then, extracts showing more than 50% quenching activity were further analyzed to determine IC_50_ values using lower concentrations.

#### Iron-Chelation Assay

The ability of plant samples to chelate iron at multiple sites is responsible for the antioxidant activity of the samples. All plant samples and their respective dilutions were prepared in methanol. The reaction mixture consists of 800 and 200 μl of plant sample. About 100 μl of FeCl_2_.H_2_O was incubated for 5 min at 37°C; then, 400 μl of 5 mM ferrozine was added and incubated for 10 min more. The absorbance was recorded at a wavelength of 562 nm by using ethylenediaminetetraacetic acid (EDTA) as a positive control standard for comparison.

#### Nitric Oxide Scavenging Assay

The nitric oxide scavenging activity of methanolic *O. limbata* was assessed by the procedure of Bhaskar and Balakrishnan (2009), which utilizes Griess reagent as the main ingredient. All test samples were prepared in dimethylsulfoxide (DMSO). The reaction mixture consists of 100 μl of plant sample and 100 μl of 10 Mm sodium nitroprusside. The reaction mixture was incubated for 3 h. About 1 ml of Griess reagent was added, and absorbance was taken at 546 nm using ascorbic acid as the positive control. Nitric oxide inhibition was calculated by using the above-mentioned formula.

#### Quantification of Total Flavonoid Content

The total flavonoid content of plant samples was measured by the aluminum chloride colorimetric method. About 500 μl of plant extract solution was mixed with 2.25 ml of distilled water and then with 150 μl of NaNO_2_ solution (5%). After 10 min, 300 μl of an AlCl_3_ solution (10%) was added; the reaction mixture was kept for 5 min and then vortexed. The mixture was thoroughly mixed and allowed to stand for another 15 min, and the 510 nm absorbance was measured versus a water blank. Results were expressed as rutin equivalents (mg rutin/g dried extract).

#### Quantification of Total Phenolic Content

The total phenolic content (TPC) of plant samples were determined by following the Folin–Ciocalteu method. Briefly, the crude extracts were reacted with of Folin–Ciocalteu reagent. After mixing the with reagent, the mixture was neutralized with sodium carbonate in double-distilled water and allowed to incubate for 60 min at room temperature. Optical density was measured at 750 nm. The test was performed in triplicates to get maximum accuracy. Results were expressed as the equivalent to milligrams of gallic acid per 100 g of dry weight.

#### Fourier Transform Infrared Spectrophotometer (FTIR)

FTIR spectrophotometer will be used to identify the characteristic functional group. The sample will be crushed with KBr, and the pellet will be developed with the help of mechanical pressure. It will observe the altered coming wavelengths in the FTIR apparatus. About 10 mg of the dried *O. limbata* powdered extract was encapsulated in 100 mg of KBr pellet, to prepare translucent sample discs. The sample was loaded in FTIR spectroscope (IR Affinity 1; Shimadzu, Japan), with a scan range from 400 to 4,000 cm^–1^ with a resolution of 4 cm (Ashokkumar and Ramaswamy, 2014).

#### Gas Chromatography–Mass Spectrometry Analysis of *Otostegia limbata*

The volatile phytoconstituents of methanolic extract of *O. limbata* was investigated using gas chromatography–mass spectrometry (GC-MS). The extract was prepared using Lisec et al. (2006). The GC/MS system (Agilent Technologies, Palo Alto, CA, United States) equipped with an auto-sampler, with an HP-5MS 5% phenylmethylsiloxane capillary column (30 m × 0.25 mm × 0.25 μm film thickness; Restek, Bellefonte, PA, United States) outfitted with an Agilent HP-5973 mass selective detector in the electron impact mode (ionization energy: 70 eV) working under the experimental conditions maintained for GC. The injection volume was 2 μl of sample. Conditions were employed with a 30 min temperature program of 80–280 at 10°C min^–1^, followed by a 10 min hold at 280°C. The injector temperature was 220°C, and the flow rate of the carrier gas (helium) was 0.8 ml/min.

#### Pharmacological Studies

A preliminary investigation was done to evaluate the antiepileptic potential of plant extracts of *O. limbata* through PTZ to induce an animal model of epilepsy in experimental mice. The efficacy of plant extract was evaluated through multiple parameters including survival rate and seizure scores (Naseer et al., 2013).

#### Chemicals

Tween 80, DMSO (dimethyl sulfoxide), normal saline (0.9% NaCl), formalin, phosphate buffer solution (PBS), pentylenetetrazole (PTZ), diazepam, NFkB ELISA kit (Santa Cruz Biotechnology, Santa Cruz, CA, United States) xylene, ethyl alcohol,% HCL, 1% ammonia, Harris’ hematoxylin solution (Sigma-Aldrich, St. Louis, MO, United States) and eosin Y, bicinchoninic acid (BCA) protein test kit, 12% SDS gel.

#### Dose Optimization

The fresh methanolic extract was prepared. Three different doses of the methanolic extract tested in PTZ induce an animal model of epilepsy at 100, 200, and 300 mg/kg. Doses were prepared using 3% DMSO and 2.5% tween, while normal saline was used for volume makeup.

#### Anticonvulsant Activity

Adult male Swiss albino mice weighing 35–45 g and ages 7–10 weeks were obtained from NIH (National Institute of Health) Islamabad. The experimental animals were kept at Laboratory Animal Research Center, Riphah International University; these animals are provided with a 12 h light/12 h dark cycle at 20–24°C, and these animals are provided with excess diet and water during the experiment. All experimental procedures were carried out according to the protocols approved by the Institutional Animal Care and Use Committee of Riphah Institute of Pharmaceutical Sciences (ref no.: REC/RIPS/2018/06) and were rigorously observed to the approved protocols. The mice were randomly divided into the following groups (*n* = 6/group): normal saline group, PTZ group, diazepam group, and treated group. Three doses (100, 200, and 300 mg/kg) of the methanolic extract of *O. limbata* were orally administered to the mice 30 min before injecting PTZ. The male mice were injected intraperitoneally with a dose of 50 mg/kg of PTZ in saline solution for 4 days. Almost 30 min after the last dose, the mice were killed and their brains were preserved in formalin solution and without formalin at −80°C until further use (Naseer et al., 2013). Convulsive behavior was observed for 30 min after each injection, and the resulting seizures were scored according to the following scale: score 0, no response; score 1, behavior arrest with trembling; score 2, motionless staring and sudden arrest in animal behavior; score 3 facial twitching; score 4 neck jerks; score 5 clonic seizure in sitting position; score 6 clonic seizure but animal does not lose its balance; score 7 tonic-clonic seizure; score 8 tonic-clonic with falling on one side; score 9 wild jumping; score 10 tonic extension, leading to death (Van Erum et al., 2019).

#### Hematoxylin and Eosin Staining

Tissue sections (*n* = 6) were fixed in 4% formaldehyde with PBS (0.1 M) and washed with water. Brain sections were dehydrated with ethyl alcohol dilutions from 70 to 100%. After that, brain tissues were washed with xylene and fixed in paraffin (Leica, Westlaw, Germany). Paraffin blocks were cut into 4 μm sections, deparaffinized with xylene, and hydrated by graded ethyl alcohol dilutions (from 100 to 70%). Sections were stained with Harris’ hematoxylin solution (Sigma-Aldrich, St. Louis, MO, United States) for 3 min and eosin Y (Sigma-Aldrich) for 1 min after that brain tissues were cleaned with water, dehydrated with graded ethyl alcohol series, mounted (Thermo Fisher Scientific, Waltham, MA, United States), and photographed using an Olympus microscope (Olympus, Tokyo, Japan).

#### Enzyme-Linked Immunosorbent Assay (ELISA)

The altered expression p-NFκB, TNF-α was assessed using mice p-NFκB and TNF-α through an enzyme-linked immunosorbent assay (ELISA) kit according to the manufacturer’s instructions. The brain tissues (*n* = 6) stored at −80°C were centrifuged at 15,000 rpm using Silent Crusher M (Heidolph) for 10 min and the supernatant was separated. The total protein concentration in each group was determined by the BCA technique (Elabscience), and an equal amount of protein was subsequently loaded to determine the concentration of p-NFκB and TNF-α by using an ELISA microplate reader (BioTek ELx808), and finally, the concentrations (pg/ml) were then standardized to total protein content (pg/mg total protein).

#### Western Blotting

For western blot analysis, brain tissue (*n* = 6) was dissolved in a buffer and centrifuged. A BCA protein test kit was used to determine protein content. About 30 μg of protein homogenate was electrophoretically separated on a 12% SDS gel and put to a polyvinylidene fluoride membrane. Membranes were incubated with primary antibodies, such as p-NFkB and TNF-α, overnight at 4°C before being blocked for 60 min at normal room temperature with 5% bovine serum albumin. After three washes with tris-buffered saline containing 0.1% Tween 20, the membranes were reacted with a 1:1,000 dilution of secondary anti-mouse antibodies at room temperature for 4 h. The immunoreactive bands were seen using an improved western blotting substrate kit. The protein expression was quantified using densitometry and ImageJ software (Zhang et al., 2019).

### Statistical Analysis

Statistical analysis was performed by using GraphPad Prism 8. The IC_50 of_ antioxidant assays were calculated by using one-way ANOVA followed by Tukey test. Neurobehavior was analyzed by one-way ANOVA. The ELISA of NF-κB was analyzed by two-way ANOVA, whereas the expression of TNF-α was analyzed by one-way ANOVA. The data of western blotting were analyzed by using one-way ANOVA. Statistical significance was set at *P* < 0:05. All data are expressed as the mean ± standard error of the mean (SEM).

## Results

### % Extract Yield (Extractive Value)

The percentage yield of the methanolic extract of *O. limbata* was 18 g per 100 g of dry powder. The% yield in hexane, chloroform, ethyl acetate, and butanol was 2, 1.5, 2, and 0.75 g of butanol extract, respectively.

### Evaluation of Crude Extracts for Antioxidant Potential

#### DPPH Radical Scavenging Activity

**FIGURE 2:**
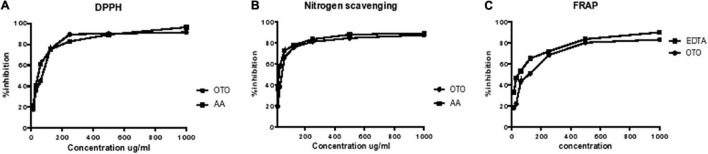
Effect of *O. limbata*: *in vitro* antioxidant assays. **(A)** DPPH percent inhibition. **(B)** Percent inhibition by iron chelation. **(C)** Nitric oxide percent scavenging.

DPPH assay is used to calculate the free-radical scavenging ability of test samples. The change in color from violet to yellow showed the scavenging activity of crude methanolic extract because DPPH gets reduced by giving a hydrogen atom. The *O. limbata* extract showed 63.1 μg/ml IC_50_, and the ascorbic acid have shown 49.76 μg/ml (Table 1A). The IC_50_ of plant extract showed that methanolic extract of *O. limbata* possesses high antioxidant activity (Figure 2A).

**TABLE 1A:** IC_50_ of antioxidant tests DPPH, FRAP, and NO scavenging of *O. limbata*.

Antioxidant test	DPPH	FRAP	No scavenging
	(IC_50_ μl/ml)	(IC_50_ μl/ml)	(IC_50_ μl/ml)
*O. limbata*	63.37	61.01	46.53
Ascorbic acid	49.76	–	23.98
EDTA	–	54.46	–

#### Iron Chelation Activity

The methanolic extract of *O. limbata* was found to be effective in chelating iron. The IC_50_ for iron chelation was 61.01 μg/ml of plant extract. The IC_50_ of standard EDTA was 54.46 μg/ml. The IC_50_ value of plant extract showed that *O. limbata* possesses high antioxidant potential (Figure 2B).

#### Nitric Oxide Scavenging Activity

The methanolic extract of *O. limbata* was tested for nitric oxide scavenging activity through IC_50_. The results showed that *O. limbata* extract possesses NO scavenging activity with IC_50_ 46.53 μg/ml (Table 1A and Figure 2C).

#### Total Phenolic Content/Total Flavonoid Content

**FIGURE 3:**
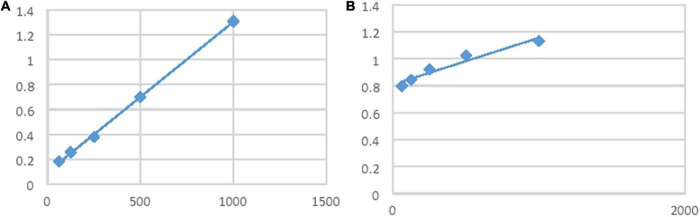
**(A)** Total phenolic content. **(B)** Total flavonoid content.

The phenolic content and flavonoid content in the plant samples were determined using a specific method. Phenolic and flavonoid compounds in the plant extract exhibited reduction properties that are responsible for antioxidants’ activities. Table 1B shows that the crude extract of *O. limbata* possesses 109 mg gallic acid/g of extract TPC and 78 mg rutin/g of extract (Figures 3A,B).

**TABLE 1B:** TPC and total flavonoid content of methanolic extract of *O. limbata.*

TPC mg gallic acid/g of extract	TFC mg rutin/g of extract
109	78

#### Fourier Transform Infrared Spectrophotometer (FTIR)

**FIGURE 4:**
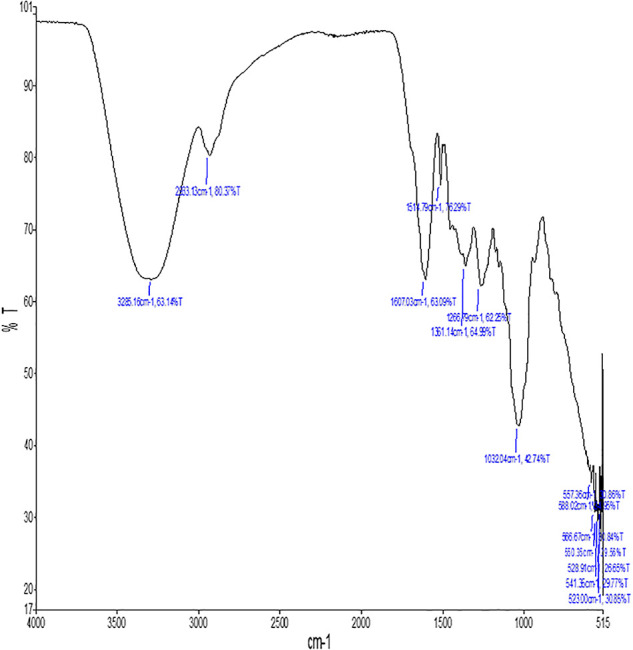
Fourier Transform Infrared Spectrophotometer (FTIR) of methanolic extract of *O. limbata*.

The FTIR spectrum of *O. limbata* was performed to recognize the functional group of the active constituents. The results of FTIR peak values and functional groups were shown in Table 2. The FTIR spectrum of *O. limbata* (Figure 4) validates the presence of phenols, alkanes, aldehyde, esters, and alcohol in the methanolic extracts of medicinal plants. The peak at 3,285 cm^–1^ indicates the presence of phenols, and the peak at 2,933 cm^–1^ refers to the presence of alkanes. The peak of 1,607 showed the presence of an aldehyde, whereas the peak of 1,361 refers to the presence of alkanes, and 1,032 showed the presence of primary alcohol (Ashokkumar and Ramaswamy, 2014; Jeyasree et al., 2014). The presence of these functional groups confirmed the presence of bioactive compounds in *O. limbata*.

**TABLE 2:** FTIR analysis of methanolic extract of *O. limbata.*

Wavenumber	Functional group	References
3,285	OH of phenols	
2,933	(C-H stretching) alkanes	Lakshmi et al., 2011
1,607	Aldehyde	Jeyasree et al., 2014
1,514	Normal aliphatic esters	Ashokkumar and Ramaswamy, 2014
1,361	(C-H) alkanes	Fernando et al., 2019
1,266	-OH of phenols	
1,032	-OH primary alcohol	

#### Gas Chromatography–Mass Spectrometry (GC-MS) of Methanolic Extract of *Otostegia limbata*

**FIGURE 5:**
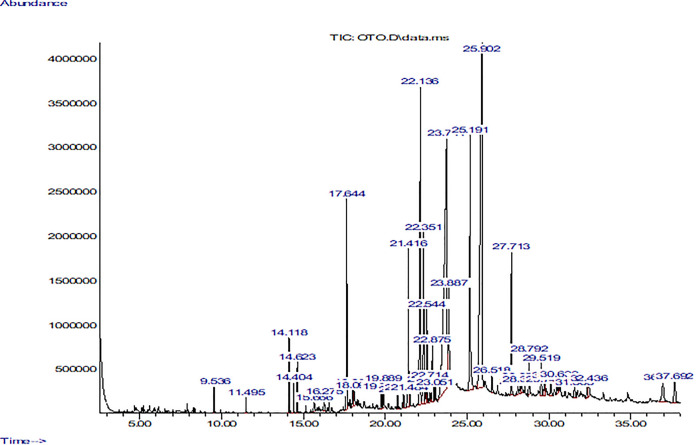
GCMS chromatogram of methanolic extract of *O. limbata*.

The GC-MS chromatogram of the methanolic extract of *O. limbata* revealed the presence of 65 compounds’ peaks. The peaks were denoted by its retention time and area% and CAS number in Table 3. Many of the compounds identified from peaks were known to possess antioxidant, anti-inflammatory, and anticancer activities (Table 3). The chromatogram showed the presence of many compounds such as (+)-4-carene, alpha-pinene, caryophyllene, *cis*-(Z)-alpha-bisabolene epoxide, pentadecanoic acid, 14-methyl-., n-hexadecanoic acid, octadecanoic acid, 2,6,10-dodecatrien-1-ol, 3,7, 11., (Z, Z)-. alpha-farnese, 1,3,3-trimethyl-2-hydroxymethyl., 2,6,10,14,18,22-tetracosahexaen, 1,2-benzenedicarboxylic acid, m., alpha-tocopherol 3,4-2H-coumarin, 4,4,5,6,8-pent and others (Figure 5). These compounds have been reported to exhibit antimicrobial, antioxidant, neuroprotective, and anticancer activities (Sabik and Abd El-Rahman, 2009; Bahi et al., 2014; Salehi et al., 2019). Many compound peaks need to be investigated for their pharmacological activities.

**TABLE 3:** GC-MS analysis of bioactive constituents of methanolic extract of *O. limbata.*

RT	Compounds	Area%	CAS No.	Medicinal properties	References
4.69	(+)-4-Carene	0.03	029050-33-7	Antimicrobial, antioxidant	Govindarajan et al., 2016
5.25	alpha-Pinene	0.10	007785-70-8	Antioxidant, anti-inflammatory, anti-*Leishmania*, anticonvulsant, and neuroprotective effects	Salehi et al., 2019
9.53	Caryophyllene	0.31	000087-44-5	Anxiety and depression	Bahi et al., 2014
15.14	Pentadecanoic acid, 14- methyl-,..	0.10	005129-60-2	Antimicrobial, antifungal	Rao et al., 2019
15.66	n-Hexadecanoic acid	0.36	000057-10-3	Anti-inflammatory activity, antioxidant	Krishnamoorthy and Subramaniam, 2014
18.33	Octadecanoic acid	0.13	000057-11-4	Antioxidant, anticancer	Wilsy et al., 2021
22.14	2,6,10-Dodecatrien-1-ol, 3,7, 11…Farnesol	8.24	000106-28-5	Anti-inflammatory, antiallergic, antioxidant, anticancer	Ku and Lin, 2015; Jung et al., 2018
22.88	(Z,Z)-alpha-Farnesene	1.54	1000293-03-1	antioxidant	Çelik et al., 2014
23.05	1,3,3-Trimethyl-2-hydroxymethyl.	0.34	1000144-10-7	Antibacterial	Peng et al., 2017
27.71	2,6,10,14,18,22-Tetracosahexaen.	3.08	000111-02-4	Anti-inflammatory, antibacterial, antitumor, immunostimulant, cancer preventive	Jenecius et al., 2012
23.89	1,2-Benzenedicarboxylic acid, m.	4.10	004376-20-9	Antifungal, antibacterial, antioxidant	Devi and Muthu, 2014; Mamun et al., 2015
30.50	3,4-2H-Coumarin, 4,4,5,6,8-pent.	0.50	1000129-70-7	Antioxidant, neuroprotective	Gaspar et al., 2015
32.34	alpha-Tocopherol	0.55	1000128-08-6	Antioxidant, neuroprotective	Sabik and Abd El-Rahman, 2009

*GC-MS analysis identified a number of compounds in O. limbata that were reported to possess pharmacological activities.*

#### Anticonvulsant Activity of the Methanolic Extract of *Otostegia limbata*

**FIGURE 6:**
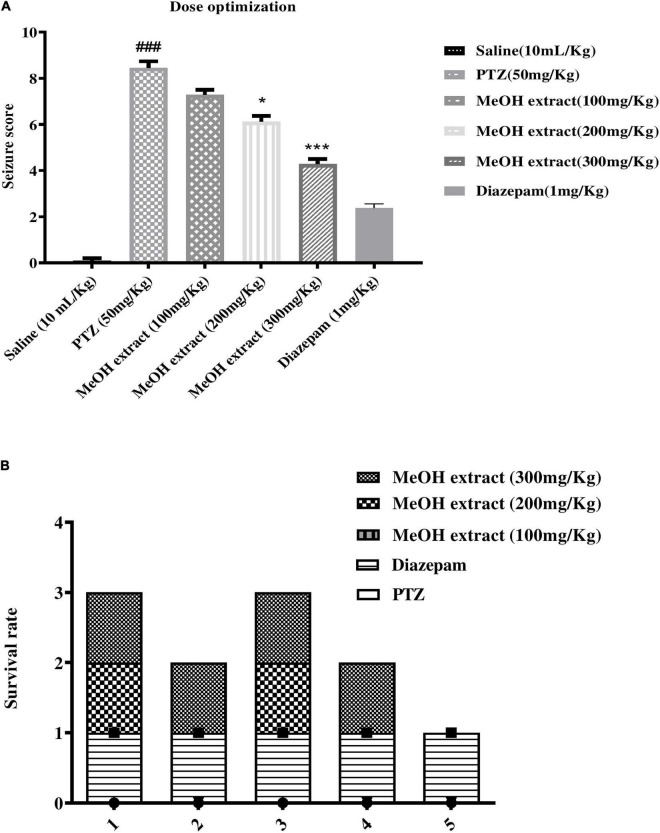
**(A)** Graph showing effect of different dosses of *O. limbata* against convulsion induced by PTZ in mice model. Symbols *indicates *p* < 0.05 and *** or ### indicates *p* < 0.001. Symbols # shows a considerable difference compared to the saline group, while *shows a considerable difference compared to the PTZ group. **(B)** Showing% mortality of mice.

To examine the antiepileptic activity of methanolic extract of *O. limbata*, male mice (35−40 g) were divided into six groups (*n* = 6). The antiepileptic potential of plant extract was determined employing PTZ-induced chemical convulsions in mice. The methanolic extract of *O. limbata* was administered 30 min before PTZ. One-way ANOVA, followed by Tukey’s multiple comparison test, was performed to analyze the results. The results showed that PTZ induced significant convulsion with a mean seizure score of 8.45 ± 0.275 (###*P* < 0.001) as compared to the saline group. Three different doses of plant extract (100, 200, and 300 mg/kg) were tested for their efficacy against PTZ-induced epilepsy. The 100 and 200 mg/kg doses could not show significant results in terms of seizure score (7.291 ± 0.203) (*P < 0.01) and latency (6.125 ± 0.232) (*P < 0.01), respectively. However, at 300 mg/kg, the plant extract showed a prominent reduction in onset and duration of colonic convulsion with a mean score (4.29 ± 0.202) (***P < 0.001). The effect was dose dependent (Figure 6).

#### Hematoxylin and Eosin Staining

**FIGURE 7:**
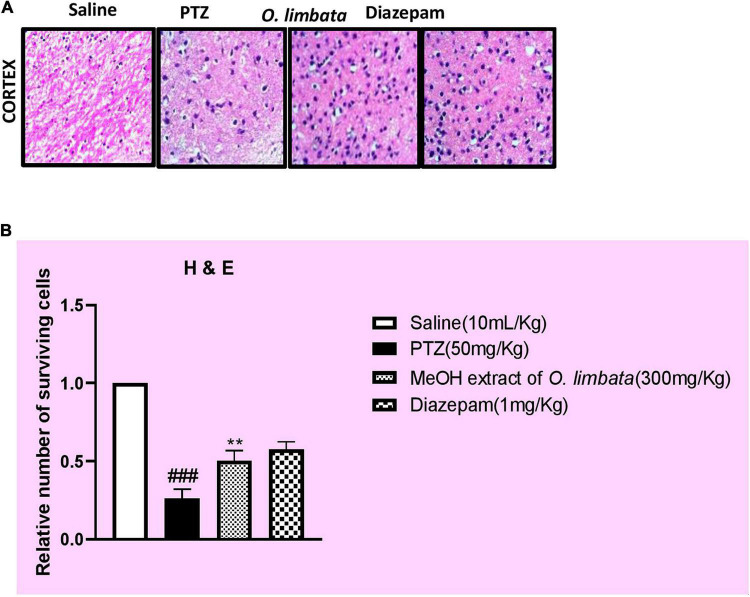
**(A)** Slides demonstrate the morphological comparison of PTZ and treated group of mice using hematoxylin and eosin (H&E) staining. The difference in number of surviving neurons is clear in graphs. **(B)** Symbols ### indicate significant difference from saline at *P* < 0.001 while symbols ** indicate significant difference from PTZ (disease) group at *P* < 0.01.

The effect of methanolic extract on the number of surviving neurons in the cortex of the mice brain using hematoxylin and eosin staining were studied. A higher number of dead neurons in the PTZ group (###*P* < 0.001) in contrast to the saline group were noted. However, the extract mitigated the number of dead neurons by an increase in the number of surviving neurons in the treated group (***P < 0.001) (Figure 7).

#### Enzyme-Linked Immunosorbent Assay

**FIGURE 8:**
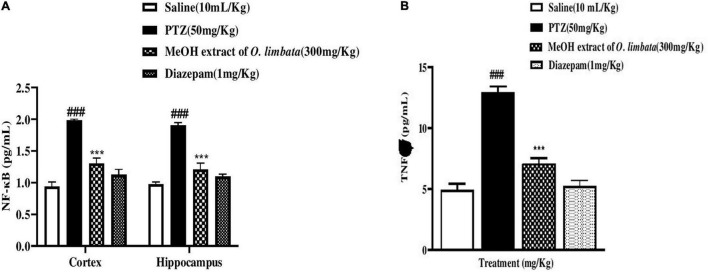
**(A)** Graphical representation of effect of *O. limbata* on the downregulated expression of p-NF-kB in the cortex and hippocampus of all treated mice; symbol ### symbolizes the significant difference from saline at *P* < 0.001, while symbols *** indicate significant difference from PTZ at *P* < 0.001. **(B)** Effect of *O. limbata* on TNF-α expression in the cortex of all treated mice; symbols. ### symbolizes the significant difference from saline at *P* < 0.001, while symbols *** indicate a significant difference from PTZ at *P* < 0.001.

The effect of *O. limbata* extract in the downregulation of the NF-κB signaling pathway and an important cytokine TNF-α in the cortex and hippocampus of mice were analyzed through ELISA. Two-way ANOVA followed by Tukey’s multiple comparisons test is shown in Figure 8. NF-κB expression was upregulated in the PTZ group (cortex ###*P* < 0.001 relative to saline group), and (hippocampus ***P < 0.001 relative to saline). Plant extract significantly downregulates the NF-κB expression in the treated group **P < 0.01 relative to PTZ control group: hippocampus, at **P < 0.01 relative to PTZ control group: plant extract treatment group: cortex, at ***P < 0.001 relative to PTZ control group, hippocampus, at ***P < 0.001 relative to PTZ control group. An important neuroinflammatory cytokine downstream of NF-κB is TNF-α. The expression of TNF-α was significantly upregulated in the PTZ control group in both cortex and hippocampus: cortex (###*P* < 0.001 relative to the saline group); hippocampus, (***P < 0.001 relative to the saline group), respectively. These results, therefore, indicate that methanolic extract of *O. limbata* reduced PTZ-induced neurodegeneration by decreasing NF-κB and downstream cytokine TNF-alpha expression in both the cortex and hippocampus of mice (Figure 8).

#### Western Blot Analysis

**FIGURE 9:**
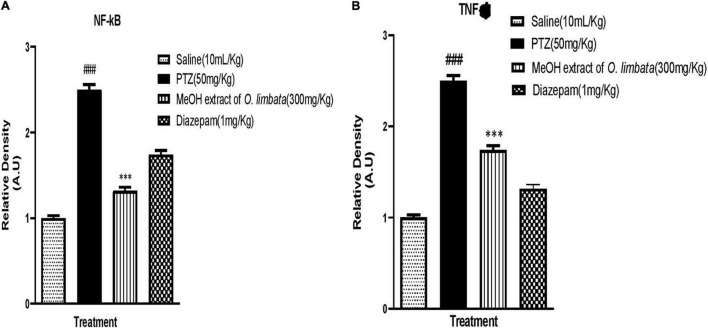
**(A)** Inhibitory effect of *O. limbata* against phosphorylated nuclear factor kappa B cells (p-NFkB) in PTZ-induced mice brain tissues, using western blotting. One-way ANOVA with post hoc Tukey’s test. Values expressed as mean ± SEM (*n* = 3). ^###^*P* < 0.0001 vs. saline group; ***P < 0.0001 vs. PTZ group. **(B)** Inhibitory effect of *O. limbata* against tumor necrosis factor alpha (TNF-α) in PTZ-induced mice brain tissues, using western blotting. One-way ANOVA with post hoc Tukey’s test and values expressed as mean ± SEM (*n* = 3). ^###^*P* < 0.0001 vs. saline group; ***P < 0.0001 vs. PTZ group.

NF-κB is responsible for controlling signaling pathways in many inflammatory diseases. The PTZ group showed a higher expression of both phosphorylated nuclear factor kappa B cells (p-NFkB) and tumor necrosis factor–alpha (TNF-α) (###*P* < 0.0001) in brain tissues, whereas the level of p-NFkB and TNF-α was downregulated in diazepam and extract-treated brain tissues (***P < 0.0001) (***P < 0.0001), respectively (Figure 9). This indicates that plant extract in treated group reduced the expression of p-NFkB and TNF-α in brain tissues.

## Discussion

Plants’ important role in brain cell protection in response to various types of traumas inflicted to brain is very fascinating. Studies have been reported where formulations with multiple phytoconstituents have proven to be therapeutically active in nervous system diseases (Tao et al., 2020). *O. persica* has been reported for anticonvulsant activity (Ansari et al., 2014). *O. limbata* is closely related to this species. The literature on its pharmacological and phytochemical investigations is very limited. In our study, for the anti-epileptic potential in the PTZ-induced model of epilepsy in rats, methanolic extract was selected. During a literature survey, we found that the methanolic extracts of multiple plants exert neuroprotective effects (Velmurugan et al., 2012; Ansari et al., 2014).

In the methanolic extract of *O. limbata*, many important bioactive compounds including phenols, flavonoids, coumarins, terpenoids, tannins, alkaloids, saponins, quinones, and glycosides were detected. Flavonoids, terpenoids, and phenols have a protective role in oxidation, inflammation, cancer, and neurological disorders (Abate and Yayinie, 2018).

DPPH, nitric oxide scavenging, and iron chelation are subtle and immediate tests to determine the antioxidant potential of plant extract. In the present study, *O. limbata* showed significant antioxidant activity. Nitric oxide scavenging is based on lower levels of nitrite and nitrate in the reaction mixture because of the consumption of oxygen by plant extract. These compounds are produced due to the breakdown of the sodium nitroprusside in the aqueous solution of Griess reagent (Kanugula et al., 2014). It is evident from the literature that many ailments can be treated by adding a high level of flavonoids in one’s daily diet. It is reported that the antioxidant potency of flavonoids is that they promote health and have a stronger ability for electron donation (Hernandez-Hernandez et al., 2009).

Several studies suggest that reactive oxygen species (ROS) play a key role in generating epilepsy and other neurological disorders. A scientific study notifies that PTZ-induced seizures are linked with increased oxide-nitrosative stress. Scientists are trying to reduce neurodegeneration by using antioxidant compounds (Lee et al., 2020). The present study depicted that *O. limbata* extract exerts its protective effect against elevated nitrosative stress (Tao et al., 2020). The presence of the functional groups of bioactive compounds was confirmed through FTIR analysis. The functional groups confirmed the presence of bioactive compounds in the methanolic extract of *O. limbata*, which was found to possess antioxidant and anti-inflammatory activity. The GC-MS analysis of *O. limbata* identified a number of volatile compounds that were reported to possess antioxidant, anti-inflammatory, and neuroprotective potential (Krishnamoorthy and Subramaniam, 2014; Salehi et al., 2019). Previous researchers suggested that the presence of limbatolide A, B, C, D limbatenolide-E, eupatorin and 3′-O-methyl eupatorin, and ballotenic and ballodiolic acids are terpenoids and flavones found in *O. limbata* (Sadaf et al., 2016). Our study focuses on the antiepileptic potential of *O. limbata* mediated through the balancing excitatory (glutamate) and inhibitory gamma-aminobutyric acid (GABA) brain monoamines and inhibition of p-NFkB/TLR-4 pathway to ameliorate neuroinflammation (TNF-a, IL-1b, and COX-2).

**FIGURE 10:**
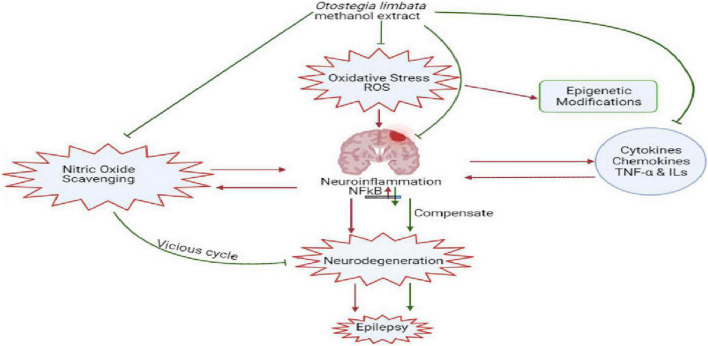
Diagrammatic representation of the role of oxidative stress and neuro inflammation in generating epilepsy and antioxidant and neuroprotective potential of limbata in suppression of inflammatory cytokines and down regulation of NFkB.

Oxidative stress and neuroinflammation due to impaired blood–brain barrier (BBB) are the leading cause of epileptogenesis, which could induce p-NFκB activation, p-NFκB regulated genes, and proinflammatory cytokines TNF-α and ILs (Shi et al., 2017). p-NFκB is responsible for controlling signaling pathways in many inflammatory diseases. However, there is a need to apprehend how the activated inflammatory pathway contributes to the pathogenesis of epilepsy. It has been reported that the overexpression of proinflammatory cytokines and chemokines affects the structure and function of neurons by damaging the BBB and neuronal hyperexcitability (Vezzani et al., 2013). TNF-α, p-NFkB signaling, and IL-1β can induce the activation of α-amino-3-hydroxyl-5-methyl-4-isoxazole-propionate (AMPA) and N-methyl-D-aspartate (NMDA) receptors, increasing AMPA- and NMDA-mediated calcium ion invasion into neurons and consequently boosting neurons’ hyperexcitation and the downregulation of GABA receptors (Chen et al., 2018). Besides, inflammation reactions may alter the BBB permeability (Yin et al., 2013). Our results of ELISA and western blotting confirmed that inflammatory markers (p-NFkB and TNF-α) showed enhanced expression in the PTZ group, whereas their expression was downregulated in *O. limbata* in the treated group (Figure 10).

It is investigated that the administration of PTZ is associated with an upregulated inflammatory response in the brain region of mice, which was inhibited by the administration of *O. limbata.* Our study may contribute to the novel identification of anti-inflammatory potential *via* the inhibition of TNF-a, using IL-1. The *O. limbata* methanolic plant extract possesses antioxidant and anti-inflammatory properties, and it can be used as a potential candidate for the treatment of neurodegenerative disorders. This study warrants a further investigation of this potential natural product on other neurodegenerative disorders, exploring further aspects through genomics and proteomics. This plant needs to be investigated for the identification and quantification of the lead compound. The lead compound needs to be tested against neurodegenerative disorders.

## Conclusion

In conclusion, the present study assessed the neuroprotective effect of the methanolic extract of *O. limbata* on PTZ-induced seizure in mice. *O. limbata* could exert a neuroprotective effect by reducing oxidative stress induced by ROS and by downregulating the overexpression of inflammatory cytokines p-NFkB and TNF-α in epilepsy. This study may contribute to the novel activity exerted by *O. limbata* as a potential candidate in drug discovery, and there is a need to further investigate and isolate lead compounds having neuroprotective effects from *O. limbata*. Our investigation may offer a certain scientific basis for the clinical application of *O. limbata* against epilepsy.

## Data Availability Statement

The original contributions presented in the study are included in the article/supplementary material, further inquiries can be directed to the corresponding author.

## Ethics Statement

The animal study was reviewed by the ethical review board of International Islamic University and approved by Chairperson of the Department.

## Author Contributions

All authors have contributed to the compiling data, manuscript writing, however, FA has conducted all experimental procedures.

## Conflict of Interest

The authors declare that the research was conducted in the absence of any commercial or financial relationships that could be construed as a potential conflict of interest.

## Publisher’s Note

All claims expressed in this article are solely those of the authors and do not necessarily represent those of their affiliated organizations, or those of the publisher, the editors and the reviewers. Any product that may be evaluated in this article, or claim that may be made by its manufacturer, is not guaranteed or endorsed by the publisher.

## References

[B1] Abate L. Yayinie M. (2018). Effect of solvent on antioxidant activity of crude extracts of Otostegia integrifolia leave. *Chem. Int.* 4 183–188.

[B2] Ansari F. Shafaroodi H. Asgarpanah J. (2014). Anticonvulsant Activity of Otostegia persica (Burm.) Boiss. *Biosci. Biotechnol. Res. Asia* 11 733–737. 10.13005/bbra/1329

[B3] Ashokkumar R. Ramaswamy M. (2014). Phytochemical screening by FTIR spectroscopic analysis of leaf extracts of selected Indian medicinal plants. *Int. J. Curr. Micro. Appl. Sci.* 3 395–406.

[B4] Bahi A. Al Mansouri S. Al Memari E. Al Ameri M. Nurulain S. M. Ojha S. (2014). β-Caryophyllene, a CB2 receptor agonist produces multiple behavioral changes relevant to anxiety and depression in mice. *Physiol. Behav.* 135 119–124. 10.1016/j.physbeh.2014.06.003 24930711

[B5] Bhaskar H. Balakrishnan N. (2009). In vitro antioxidant property of laticiferous plant species from western ghats Tamilnadu, India. *Int. J. Health Res.* 2 163–170. 10.4314/ijhr.v2i2.55413

[B6] Brand-Williams W. Cuvelier M. E. Berset C. L. W. T. (1995). Use of a free radical method to evaluate antioxidant activity. *LWT Food Sci. Technol.* 28 25–30. 10.1016/S0023-6438(95)80008-5

[B7] Çelik K. Toğar B. Türkez H. Taşpinar N. (2014). In vitro cytotoxic, genotoxic, and oxidative effects of acyclic sesquiterpene farnesene. *Turk. J. Biol.* 382 253–259. 10.3906/biy-1309-55 31411186

[B8] Chen L. Deng H. Cui H. Fang J. Zuo Z. Deng J. (2018). Inflammatory responses and inflammation-associated diseases in organs. *Oncotarget* 9 7204–7218. 10.18632/oncotarget.23208 29467962 PMC5805548

[B9] Devi J. Muthu A. K. (2014). Gas chromatography-mass spectrometry analysis of bioactive constituents in the ethanolic extract of Saccharum spontaneum Linn. *Int. J. Pharm. Pharm. Sci.* 6 755–759.

[B10] Fernando L. M. Biswal A. R. Pazhamalai V. (2019). Phytochemical, FTIR and NMR analysis of crude extract of Acacia planifrons seeds. *J Pharm. Sci. Res.* 11 1960–1962.

[B11] Gaspar A. Milhazes N. Santana L. Uriarte E. Borges F. Matos M. J. (2015). Oxidative stress and neurodegenerative diseases: looking for a therapeutic solution inspired on benzopyran chemistry. *Curr. Top. Med. Chem.* 15 432–445. 10.2174/1568026614666141229124141 25658803

[B12] Govindarajan M. Rajeswary M. Benelli G. (2016). δ-Cadinene, calarene and δ-4-carene from Kadsura heteroclita essential oil as novel larvicides against malaria, dengue and filariasis mosquitoes. *Comb. Chem. High Throughput Screen.* 19 565–571. 10.2174/1386207319666160506123520 27151483

[B13] Hernandez-Hernandez E. Ponce-Alquicira E. Jaramillo-Flores M. E. Legarreta G. L. (2009). Antioxidant effect of rosemary (Rosmarinus officinalis L) and oregano (Origanum vulgare L) extracts on TBARS and color of model raw batters. *Meat Sci.* 81 410–417. 10.1016/j.meatsci.2008.09.004 22064182

[B14] Ishola I. O. Olayemi S. O. Yemitan O. K. Ekpemandudiri N. K. (2013). Mechanisms of anticonvulsant and sedative actions of the ethanolic stem-bark extract of Ficus sur Forssk (Moraceae) in rodents. *Pak. J. Biol. Sci.* 16 1287–1294. 10.3923/pjbs.2013.1287.1294 24511736

[B15] Jenecius A. Uthayakumaria F. Mohan V. R. (2012). GC-MS determination of bioactive components of Sauropus bacciformis blume (Euphorbiaceae). *J. Curr. Chem. Pharm. Sci.* 2 347–358.

[B42] Jeyasree J. Jenifer S. Priya S. Sukumaran V. Kezia Laveena D. (2014). HPLC spectral analysis of phytochemicals in *Solanum nigrum* L. and target protein identification. *World J. Pharm. Pharm. Sci.* 3 1182–1192.

[B16] Jung Y. Y. Hwang S. T. Sethi G. Fan L. Arfuso F. Ahn K. S. (2018). Potential anti-inflammatory and anti-cancer properties of farnesol. *Molecules* 23:2827. 10.3390/molecules23112827 30384444 PMC6278318

[B43] Kanugula A. K. Gollavilli P. N. Vasamsetti S. B. Karnewar S. Gopoju R. Ummanni R. (2014). Statin-induced inhibition of breast cancer proliferation and invasion involves attenuation of iron transport: intermediacy of nitric oxide and antioxidant defense mechanisms. *FEBS J.* 281 3719–3738. 24964743 10.1111/febs.12893

[B17] Kasote D. M. Katyare S. S. Hegde M. V. Bae H. (2015). Significance of antioxidant potential of plants and its relevance to therapeutic applications. *Int. J. Biol. Sci.* 11:982. 10.7150/ijbs.12096 26157352 PMC4495415

[B18] Khan S. Syed F. (2013). Bioactive constituents from genus Otostegia. *SARJ Phys. Sci.* 1 15–25.

[B19] Krishnamoorthy K. Subramaniam P. (2014). Phytochemical profiling of leaf, stem, and tuber parts of Solena amplexicaulis (Lam.) Gandhi using GC-MS. *Int. sch. Res. Notices* 2014:567409. 10.1155/2014/567409 27379314 PMC4897340

[B20] Ku C. M. Lin J. Y. (2015). Farnesol, a sesquiterpene alcohol in herbal plants, exerts anti-inflammatory and antiallergic effects on ovalbumin-sensitized and-challenged asthmatic mice. *Evid Based Complement Alternat. Med.* 2015:387357. 10.1155/2015/387357 25960750 PMC4417576

[B21] Lakshmi A. Singh S. Mehta A. (2011). Antimicrobial screening of methanol and aqueous extract of Swertia chirata. *Int. J. Pharm. Pharm. Sci.* 3 142–146.

[B49] Lee K. H. Cha M. Lee B. H. (2020). Neuroprotective effect of antioxidants in the brain. *Int. J. Mol. Sci.* 21:7152. 10.3390/ijms21197152 32998277 PMC7582347

[B50] Lisec J. Schauer N. Kopka J. Willmitzer L. Fernie A. R. (2006). Gas chromatography mass spectrometry–based metabolite profiling in plants. *Nat. Protoc.* 1 387–396. 10.1038/nprot.2006.59 17406261

[B22] Mamun M. I. R. Abd El-Aty A. M. Rahman M. M. Choi J. H. Yun K. W. Shin H. C. (2015). Characterization of secondary metabolite compounds correlated with the seasons in Artemisia princeps var. orientalis (Pamp.) H. hara leaves using direct sample injection and gas chromatography–mass spectrometry: contribution to phytotoxicity. *J. Korean Soc. Appl. Biol. Chem.* 58 173–183. 10.1007/s13765-015-0020-3

[B23] Naseer M. I. Ullah I. Al-Qahtani M. H. Karim S. Ullah N. Ansari S. A. (2013). Decreased GABA BR expression and increased neuronal cell death in developing rat brain after PTZ-induced seizure. *Neurol. Sci.* 34 497–503. 10.1007/s10072-012-1083-0 22484544

[B24] Peng W. Li D. Zhang M. Ge S. Mo B. Li S. (2017). Characteristics of antibacterial molecular activities in poplar wood extractives. *Saudi J. Biol. Sci.* 24 399–404. 10.1016/j.sjbs.2015.10.026 28149179 PMC5272933

[B44] Phaniendra A. Jestadi D. B. Periyasamy L. (2015). Free radicals: properties, sources, targets, and their implication in various diseases. *Indian J. Clin. Biochem.* 30 11–26. 10.1007/s12291-014-0446-0 25646037 PMC4310837

[B45] Rao M. R. Anisha G. S. Prabhu K. V. Shil S. Vijayalakshmi N. (2019). Preliminary phytochemical and gas chromatography-mass spectrometry study of one medicinal plant *Carissa carandas*. *Drug Invent. Today* 12 1629–1630.

[B25] Sabik L. M. Abd El-Rahman S. S. (2009). Alpha-tocopherol and ginger are protective on Cyclophosphamide-induced gonadal toxicity in adult male albino rats. *Basic Appl. Pathol.* 2 21–29. 10.1111/j.1755-9294.2009.01034.x

[B46] Sadaf H. M. Bibi Y. Riaz Y. Saboon Sultan M. S. Bibi F. (2016). Pharmacological aptitude and profiling of active constituent from *Otostegia limbata*-comprehensive review. *Asian Pac. J. Trop. Dis.* 6 918–924.

[B26] Salehi B. Upadhyay S. Erdogan Orhan I. Kumar Jugran A. L D Jayaweera S. A Dias D. (2019). Therapeutic Potential of α- and β-Pinene: a Miracle Gift of Nature. *Biomolecules* 9:738. 10.3390/biom9110738 31739596 PMC6920849

[B27] Sasidharan S. Chen Y. Saravanan D. Sundram K. M. Latha L. Y. (2011). Extraction, isolation and characterization of bioactive compounds from plants’ extracts. *Afr. J. Tradit. Complement. Altern. Med.* 8 1–10. 10.4314/ajtcam.v8i1.60483 PMC3218439 22238476

[B28] Scharfman H. E. (2007). The neurobiology of epilepsy. *Curr. Neurol. Neurosci. Rep.* 7 348–354. 10.1007/s11910-007-0053-z 17618543 PMC2492886

[B29] Scorza C. A. Marques M. J. da Silva S. G. da Graça Naffah-Mazzacoratti M. Scorza F. A. Cavalheiro E. A. (2018). Status epilepticus does not induce acute brain inflammatory response in the Amazon rodent Proechimys, an animal model resistant to epileptogenesis. *Neurosci. Lett.* 668 169–173. 10.1016/j.neulet.2017.02.049 28235602

[B30] Shekh-Ahmad T. Kovac S. Abramov A. Y. Walker M. C. (2019). Reactive oxygen species in status epilepticus. *Epilepsy Behav.* 101:106410. 10.1016/j.yebeh.2019.07.011 31378559

[B47] Shi L. M. Chen R. J. Zhang H. Jiang C. M. Gong J. (2017). Cerebrospinal fluid neuron specific enolase, interleukin-1ß and erythropoietin concentrations in children after seizures. *Childs Nerv. Syst.* 33 805–811. 28236069 10.1007/s00381-017-3359-4

[B31] Siddiqui F. Sultan T. Mustafa S. Siddiqui S. J. Ali S. Malik A. (2015). Epilepsy in pakistan: national guidelines for clinicians. *Pak. J. Neurol. Sci.* 10 47–62.

[B32] Tao Z. Chun-Yan H. Hua P. Bin-Bin Y. Xiaoping T. (2020). Phyllathin from Phyllanthus Amarus Ameliorates Epileptic Convulsion and Kindling Associated Post-Ictal Depression in Mice *via* Inhibition of NF-κB/TLR-4 Pathway. *Dose Response* 18:1559325820946914. 10.1177/1559325820946914 32821254 PMC7412921

[B33] Truelove S. Zhu H. Lessler J. Riley S. Read J. M. Wang S. (2016). A comparison of hemagglutination inhibition and neutralization assays for characterizing immunity to seasonal influenza A. *Influenza Other Respir. Viruses* 10 518–524. 10.1111/irv.12408 27406695 PMC5059953

[B34] Van Erum J. Van Dam D. De Deyn P. P. (2019). PTZ-induced seizures in mice require a revised Racine scale. *Epilepsy Behav.* 95 51–55. 10.1016/j.yebeh.2019.02.029 31026782

[B35] Velmurugan V. Arunachalam G. Ravichandran V. (2012). Anticonvulsant activity of methanolic Extract of Prosopis cineraria (Linn) Druce stem barks. *Int. J. Pharmtech Res.* 4 89–92.

[B36] Vezzani A. Friedman A. Dingledine R. J. (2013). The role of inflammation in epileptogenesis. *Neuropharmacology* 69 16–24. 10.1016/j.neuropharm.2012.04.004 22521336 PMC3447120

[B37] Visweswari G. Prasad K. S. Chetan P. S. Lokanatha V. Rajendra W. (2010). Evaluation of the anticonvulsant effect of *Centella asiatica* (gotu kola) in pentylenetetrazol-induced seizures with respect to cholinergic neurotransmission. *Epilepsy Behav.* 17 332–335. 10.1016/j.yebeh.2010.01.002 20144879

[B38] Vuong C. Yeh A. J. Cheung G. Y. Otto M. (2016). Investigational drugs to treat methicillin-resistant Staphylococcus aureus. *Expert Opin. Investig. Drugs* 25 73–93. 10.1517/13543784.2016.1109077 26536498 PMC5004000

[B39] Wilsy J. I. Beschi D. A. Appavoo M. R. Wilsy J. I. (2021). GC-MS analysis, collected from Kavalkinaru area, Tirunelveli District, Tamil Nadu, India. *Eur. J. Mol. Clin. Med.* 7 4287–4292.

[B40] Yin Y. H. Ahmad N. Makmor-Bakry M. (2013). Pathogenesis of epilepsy: challenges in animal models. *Iran. J. Basic Med. Sci.* 16:1119. PMC3909622 24494063

[B48] Yu Q. Holloway H. W. Utsuki T. Brossi A. Greig N. H. (1999). Synthesis of novel phenserine-based-selective inhibitors of butyrylcholinesterase for Alzheimer’s disease. *J. Med. Chem.* 42 1855–1861. 10.1021/jm980459s 10346939

[B41] Zhang L. Guo Z. Wang Y. Geng J. Han S. (2019). The protective effect of kaempferol on heart *via* the regulation of Nrf2, NF-κβ, and PI3K/Akt/GSK-3β signaling pathways in isoproterenol-induced heart failure in diabetic rats. *Drug Dev. Res.* 80 294–309. 10.1002/ddr.21495 30864233

